# Intermarriage of Cousins

**Published:** 1908-06-06

**Authors:** 


### Intermarriage of Cousins.

Public opinion is notoriously peculiar in its manifestations. Sometimes it takes centuries to alter its beliefs, at other times it will as readily extemporise lifelong convictions. At the present day in Europe the tendency is to discourage consanguineous marriages, though there has not always been unanimity on this subject. Persia and Phœnicia, even Greece, permitted and encouraged such marriages, while the Jews have forbidden them by law from the earliest times. So far as we know, the objections seem to have been based on social rather than scientific grounds. The appeal which Professor Karl Pearson has recently made for information and facts relating to marriages of first cousins in families, and the publication of a pamphlet discussing the degree of resemblance between such cousins, cannot fail to awaken a widespread interest. Questions of such a frankly biological aspect possess a peculiar fascination. They appeal both to the expert and to the nebulous Man-in-the-street.

It is to be hoped, therefore, that this appeal will not go unregarded. Whether statistical laws can be applied to individual cases with approximate accuracy does not as yet matter. Upon the broadest investigation, on the lines propounded by the workers in the Eugenics Laboratory, it may be possible to base a system of State regulation in the matter of marriage, if such be deemed necessary. If it were found, for example—a pure hypothesis—that the inter-marriage of consumptives tended to produce consumptive children in over 50 per cent. of cases, it would be monstrous for the State to remain indifferent. In the present venture, however, Professor Pearson is not very happy. His data (the investigation of some 300 families), we think, are insufficient, and the pamphlet is premature. The authors, Karl Pearson and Miss Elderton, however, state that "they do not consider that their data show any difference between the inheritance of physical, psychological, and pathological characters which could not be accounted for (*a*) by the difficulty of appreciating temperament and (*b*) the incompleteness of the cousin record." Though the actual entity of disease is still an academic problem, few people can be practically in doubt about it. Had the Professor confined himself to pathological and physical characters, which can be more safely estimated, the personal equation would have been less conspicuous. Who can really judge temperament?

The result of the investigation was the conclusion that the resemblance between cousins is about 0.27, and that they must in this respect be reckoned in the same category as uncles and nieces, grandparents and grandchildren. The law which forbids marriage between uncle and niece must logically forbid marriage between first cousins. In the former case there is also the difference in age, which would generally be considerable, and is an additional objection. The medical man must have another burden laid upon his shoulders, and in future must study his patients collaterally as well as directly in the matter of descent. That public attention should be called to such a serious problem is good. But it must be remembered that the problem is almost entirely biological. Inbreeding, as Charles Darwin said, tends to bring out the salient features of a family. The "gametes," to use a Mendelian catchword, are obviously more pure, and the type may be considerably strengthened by such methods, though prolonged inbreeding seems to produce a decrease of fertility. Such was the conclusion of Weismann, who inbred mice for twenty-nine generations.

Perhaps the best attitude, and the one which will appeal to medical men, is summed up in the discreet sentences of Delage: "Consanguinity adds up (*additionne*) tendencies generally. In itself it can neither be advantageous nor harmful. Everything depends on the condition of those who practise such marriages. Many authenticated examples show that they are compatible with the perfect conservation of all the qualities of the race, and the opinion which tends to prevail is that they concentrate only the vicious tendencies, and that where no constitutional weakness is, there no evil arrives." It is to be hoped that the researches of Professor Pearson will help to confirm this cheerful yet sound optimism.

